# Risk Factors Associated with Complications in Fixed Partial Dentures

**DOI:** 10.3390/jcm15041471

**Published:** 2026-02-13

**Authors:** Diana-Elena Vlăduțu, Vlad Pârvănescu, Alexandru Ștefârță, Virginia Maria Rădulescu, Maria Filoftea Mercuț, Veronica Mercuț, Monica-Mihaela Iacov-Crăițoiu, Maria Alexandra Rădoi, Antonia Samia Khaddour, Ioana Mitruț, Mihaela Roxana Brătoiu

**Affiliations:** 1 Department of Prosthetic Dentistry, University of Medicine and Pharmacy of Craiova, 200349 Craiova, Romania; diana.vladutu@umfcv.ro (D.-E.V.); alexandru.stefarta@umfcv.ro (A.Ș.); veronica.mercut@umfcv.ro (V.M.); monica.craitoiu@umfcv.ro (M.-M.I.-C.); alexandra.radoi@umfcv.ro (M.A.R.); ioana.mitrut@umfcv.ro (I.M.);; 2 Department of Plastic Surgery, University of Medicine and Pharmacy of Craiova, 200349 Craiova, Romania; vlad_parvanescu@yahoo.com; 3 Department of Medical Informatics and Biostatistics, Faculty of Medicine, University of Medicine and Pharmacy of Craiova, 200349 Craiova, Romania; 4 Department of Ophthalmology, University of Medicine and Pharmacy of Craiova, 200349 Craiova, Romania; 5 Department of Oral Rehabilitation, University of Medicine and Pharmacy of Craiova, 200349 Craiova, Romania; antoniasamia11@gmail.com

**Keywords:** fixed partial dentures, DMFT index, bruxism, smoking status, oral hygiene, FPD complications

## Abstract

**Background/Objectives**: Fixed partial dentures (FPD) are intended to restore the impaired functions of the dento-maxillary system (DMS) following the onset of partial edentulism. The objectives of this study were to determine the association of risk factors represented by caries predisposition (DMFT index - decayed (D), missing (M), and filled (F) teeth), oral hygiene, bruxism and smoking with FPD complications. **Methods**: This retrospective cross-sectional study was conducted on a sample of 428 patients who presented to the Department of Prosthetic Dentistry at the University of Medicine and Pharmacy of Craiova for various complications related to FPD. Biological, technical, and aesthetic complications were identified in terms of their frequency and their association with risk factors, including caries predisposition quantified by the DMFT index, oral hygiene, bruxism, and smoking status. Associations were assessed using Pearson’s chi-square tests and Mann–Whitney U tests (two-sided, α = 0.05), performed in IBM SPSS Statistics, version 26 (IBM Corp., Armonk, NY, USA). **Results**: The DMFT index showed elevated values, 16.41 ± 4.43 in subjects aged ≤45 years and 18.39 ± 4.89 in subjects aged >45 years. Oral hygiene was satisfactory in a percentage of 75.82%, bruxism was present in 38%, and 40.52% were smokers. Regarding FPD complications, 94.77% were biological, 86.27% were technical, and 85.62% were aesthetic. **Conclusions**: Caries predisposition, assessed by the DMFT index, and smoking were associated with a higher frequency of biological complications. Bruxism and smoking were associated with technical complications, while unsatisfactory oral hygiene and smoking were associated with aesthetic complications.

## 1. Introduction

The loss of one or more teeth at the level of the dental arches leads to a morphofunctional imbalance with repercussions affecting all components of the dento-maxillary system (DMS). Missing teeth may be replaced with fixed partial dentures (FPD), removable prostheses, or implant-supported restorations [1]. Currently, dental treatments are primarily oriented toward conservative dental care within the framework of a multidisciplinary approach [2]. Sailer et al. stated that the clinical decision regarding the various types of restorations is based on anatomical, aesthetic, and economic factors and, most importantly, on the patient’s preferences [1]. However, beyond the local and general anatomical factors related to the patient’s presenting clinical condition, it is essential to consider the functional loads acting on the future restoration [3].

The Glossary of Prosthodontic Terms defines the treatment plan as “the sequence of procedures planned for the treatment of a patient following the establishment of the diagnosis” [4]. Oral hygiene, smoking status, caries susceptibility, and periodontal health, some parafunctional habits [5] are also factors that influence the prognosis of FPD [6].

According to Goldstein, the longevity of dental restorations depends on several factors, including the materials used, patient-related factors, and dentist-related factors [7]. Gordan et al. attributed the success of prosthetic restorations to the following factors: the quality of the restoration at the time of placement, the type and size of the restoration, the material used, and the clinician’s ability to diagnose secondary caries [8]. Rosenstiel stated that a number of systemic and local factors must be considered in prosthetic treatment planning. Accordingly, prosthetic treatment planning should take into account the patient’s personality, mouth opening capacity and the health status of the temporomandibular joint (TMJ), the presence of bruxism, oral hygiene status, smoking, the caries status of the teeth, periodontal status, and the smile line [6].

The reliability of prosthetic treatment may be compromised precisely due to noncompliance with functional and occlusal requirements, with many prosthetic failures being caused by unsatisfactory occlusal adaptation of prosthetic restorations [9].

Throughout life, incisal and occlusal surfaces most often require restoration as a result of various pathological changes. When occlusal discrepancies exist between the original morphology of the dental arches and the prosthetic restoration, they frequently lead to restoration failure, manifesting as fractures, tooth wear, periodontal complications, decementation of restorations, or exacerbation of temporomandibular disorder (TMD) symptoms [10,11].

In a review, Patel et al. defined the concepts of survival, success, and failure of FPDs. Accordingly, the success of an FPD may be defined as a prosthetic restoration in which neither the framework nor the veneering material exhibits fractures, and in which there is no evidence of caries, periodontal involvement (bleeding on probing), or signs of endodontic pathology [12,13]. FPD survival may be defined as the situation in which, from the time of cementation until subsequent examination, ceramic veneer fracture, decementation of the restoration, or aesthetic changes are observed [12,14], whereas failure may be defined as “any design or technology-related defect that needs replacement of the FPD” [12,15]. The studies included by Patel et al. in this review assessed the biological, technical, and aesthetic complications of FPDs [12]. Biological complications included pain, extensive cervical caries, pulpal pathology, periapical complications, and damage to antagonist teeth. Technical complications comprised porcelain veneer fracture, abutment tooth fracture, pontic fracture, loss of retention, and loss of marginal adaptation, while aesthetic complications included compromised aesthetic and gingival recession [12].

The objectives of the present study were to determine the frequency of biological, technical, and aesthetic complications of FPDs in a group of patients and to identify the role of selected risk factors involved in their occurrence, with particular emphasis on the influence of oral hygiene, caries susceptibility, bruxism and smoking. This study represents a further investigation of a previous study conducted on the same patient group, in which complications of FPD associated with occlusal overload were evaluated in terms of their severity [16].

The null hypotheses assumed that the occurrence of biological complications in patients rehabilitated with FPDs is not associated with DMFT (decayed (D), missing (M), and filled (F) teeth), oral hygiene status, “probable” bruxism, or smoking (H0_BIO); technical complications are not associated with DMFT, oral hygiene status, “probable” bruxism, or smoking (H0_TECH); and aesthetic complications are not associated with DMFT, oral hygiene status, “probable” bruxism, or smoking (H0_AEST).

## 2. Materials and Methods

### 2.1. Study Design

This retrospective cross-sectional study was conducted between 10 January 2023, and 30 December 2024, on a group of 428 patients who presented to the Department of Prosthodontics at the University of Medicine and Pharmacy of Craiova with various complications related to FPDs. In order to achieve the study objectives, a study group consisting of patients presenting FPDs with complications was established. The complications were analyzed in relation to the characteristics of the FPDs and the associated risk factors. This study was approved by the University Ethics and Deontology Committee of UMFCV (No. 234, 7 December 2022) and was conducted in accordance with the Declaration of Helsinki [17]. Prior to participation, written informed consent was obtained from all study participants.

### 2.2. Patients’ Selection

The inclusion criteria for the study were: patients of both genders who presented with at least one conventional FPD with complications and who provided written informed consent for participation in the study.

The exclusion criteria were: patients who did not provide consent for inclusion in the study, patients with incomplete treatment records, edentulous patients or those restored with a different type of prosthesis, and patients with communication or neuromuscular coordination impairments.

### 2.3. Patient Examination

After taking the medical history, patients underwent a clinical examination, a panoramic radiograph was obtained, and the treatment records from the clinic’s database were reviewed. Intraoral examination was performed using the standard techniques of inspection, palpation, percussion, and probing, in accordance with the World Health Organization standards and the Declaration of Helsinki [17,18]. Decayed teeth, missing teeth and filled teeth were recorded. For the calculation of the DMFT index, a total of 28 teeth per arch was considered [19].

The FPDs were examined at the level of the abutment teeth and the pontic to determine the type of complication. Radiographic evaluation based on panoramic imaging was performed by the same specialist dentist.

### 2.4. Data Collection and Definition of Variables

General patient data were recorded, including gender, age, and area of residence. Regarding age, two groups were considered: individuals born before 1980, who primarily received traditional treatment methods and had limited access to dental care (age group > 45 years), and individuals ≤ 45 years, who had easier access to dental treatment and benefited from modern treatment methods. The two gender categories were male and female, while the area of residence was classified as urban or rural.

Data collected regarding the FPDs included the type of FPDs based on the material used, the number of elements, and the age of the restoration. Variables defined from these data included FPD age (clinical function of the FPDs), categorized as 0–5 years, 6–10 years, 11–15 years, 16–20 years, and over 20 years; and the total number of elements in a FPD, classified into four categories: short-span restorations (2 elements), medium-span restorations (3–4 elements), long-span restorations (5–7 elements, corresponding to a hemiarch), and extensive restorations covering multiple sextants or the entire arch (8–16 elements).

Among the local risk factors for FPD complications, caries susceptibility based on the DMFT index, oral hygiene, bruxism, and smoking were evaluated.

The DMFT index of the study participants was calculated as the sum of decayed (D), missing (M), and filled (F) teeth [20]. DMFT was determined according to the age groups included in the study. Data regarding “probable” bruxism were collected using a self-reported questionnaire that had previously been validated and used in our studies, along with a clinical examination [21]. The same questionnaire included a question regarding smoking. Two categories were defined: smoker and non-smoker (never or occasional smoker).

Oral hygiene was assessed based on the number of toothbrushing sessions per day, with two categories: satisfactory hygiene, corresponding to two or more brushing sessions per day, and unsatisfactory hygiene, corresponding to one brushing session per day or less [22].

For the evaluation of FPD complications, the classification proposed by Patel et al. was used. This classification considers biological complications (pain, extensive cervical caries, pulpal pathology, periapical complications, and damage to antagonist teeth); technical complications (fracture of veneering porcelain, abutment tooth fracture, pontic fracture, loss of retention, and loss of marginal adaptation); and aesthetic complications (compromised aesthetic and gingival recession) [12].

The data collected were entered into Microsoft Excel and statistically analyzed.

### 2.5. Statistical Analysis

The initial dataset was compiled and verified using Microsoft Excel 365 (Microsoft Corp., Redmond, WA, USA), where variables were coded, cleaned, and structured into analytic categories. All statistical analyses were subsequently conducted using IBM SPSS Statistics, version 26 (IBM Corp., Armonk, NY, USA).

Categorical variables were summarised as absolute and relative frequencies, using non-missing values as denominators. Continuous variables, including the DMFT index, were described by both mean ± standard deviation (SD) and median with interquartile range (IQR), in order to reflect central tendency and dispersion in potentially skewed distributions. Inferential analyses were structured around two predictor groups: sociodemographic and prosthetic factors (gender, age, living environment, restoration type, number of elements, and restoration age) and host-related oral health and behavioural factors (DMFT index, oral hygiene, bruxism, and smoking), each examined in relation to the three complication domains (biological, technical, and aesthetic).

Group-level associations between categorical predictors and binary complication outcomes were evaluated using Pearson’s chi-square test of independence (two-sided), with Cramér’s V reported as an effect size. Comparisons between continuous variables and binary outcomes (e.g., restoration age, DMFT vs. presence/absence of each complication type) used the Mann–Whitney U test (two-sided), with rank-biserial r as a measure of effect size. A two-sided α = 0.05 was considered statistically significant. *p*-values are reported to three decimals, or as “<0.001” when appropriate.

## 3. Results

### 3.1. Characteristics of the Study Group

After applying the inclusion and exclusion criteria, as previously described [16], the study group consisted of 306 patients, 67.97% being female and 32.03% male. Younger adults (≤45 years) represented 11.11% of the study group, while 88.89% were over 45 years of age. The mean age of the study group was 57.67 ± 8.66 years old. Regarding the area of residence, 75.16% came from urban areas, with the remaining 24.84% from rural areas.

Based on the material of the FPDs, 58.82% were metal–ceramic and 41.18% were metal–acrylic. In terms of the number of elements, 56.86% were medium-span FPDs (3–4 elements), 30.07% were long-span FPDs (5–7 elements), 10.46% were extensive FPDs (8–16 elements), and 2.61% were short-span FPDs (2 elements). Regarding the FPD age, 7.84% of FPDs had been in function for 0–5 years, 43.14% for 6–10 years, 18.95% for 11–15 years, 24.84% for 15–20 years, and 5.23% had been in function for over 20 years.

### 3.2. Local Risk Factors—Caries Susceptibility (DMFT Index), Oral Hygiene, Bruxism and Smoking Status

Caries susceptibility was high, with a mean DMFT of 18.17 ± 4.87, reflecting a substantial cumulative burden of dental disease consistent with the restorative needs of patients requiring FPDs.

The ≤45-year group showed significantly lower DMFT values than the >45-year group (16.41 ± 4.43 vs. 18.39 ± 4.89; *p* = 0.024), although the difference was modest. Most patients (75.82%) had satisfactory oral hygiene.

“Probable” bruxism was reported in 38.56% of cases, and 40.52% of patients were smokers, providing the clinical context for complication development (Table 1).

### 3.3. Evaluation of FPDs Complications—Biological, Technical, and Aesthetic

Biological complications of FPDs were the most frequent, affecting 290 patients (94.77%). Technical complications were recorded in 264 patients (86.27%), while aesthetic complications were observed in 262 patients (85.62%) (Table 2).

The observed biological, technical, and aesthetic complications were consistent with the clinical profile of FPDs. Among biological complications, pain (45.09%) and cervical caries (40.52%) were most common, followed by pulpal pathology (28.10%) and apical periodontitis (26.14%). Damage to antagonist teeth affected about half of the patients (50.32%) (Table 3).

Technical complications were less frequent, including veneer fracture (11.76%), abutment fracture (15.03%), and pontic fracture (5.22%). However, interface-related issues were common, with loss of retention in 32.67% and loss of marginal adaptation in 84.96% of FPDs (Table 3).

Recorded aesthetic complications included compromised aesthetic in 262 FPDs (85.62%) and gingival recession in 290 FPDs (94.77%) (Table 3).

Overall, these findings support the notion that, over the long term, the effects of FPD age, oral hygiene, smoking, and residential environment accumulate; when combined, biological, technical, and aesthetic complications typically occur together rather than in isolation.

### 3.4. Bivariate Associations Between Sociodemographic Factors, FPD Characteristics, and Biological, Technical, and Aesthetic Complications

Bivariate associations were performed to analyze how each complication (biological, technical, and aesthetic) relates to two categories of factors: sociodemographic factors and FPD characteristics on one hand, and local risk factors on the other. Categorical associations were evaluated using chi-square tests, while continuous variables, such as FPD age and the DMFT index, were analyzed using Mann–Whitney tests with rank-biserial effect sizes.

#### 3.4.1. Bivariate Associations Between Sociodemographic Factors, FPD Characteristics, and Biological Complications

Biological complications showed limited sociodemographic differences but similar distribution patterns across groups. Gender and age group were not statistically significant, although prevalence was high in all categories (96.15% in females vs. 91.84% in males; 100% in patients aged ≤45 years vs. 94.12% in those > 45 years). Statistically significant differences were observed for area of residence and FPD type. Biological complications were more frequent in patients from rural areas compared to those from urban areas (100% vs. 93.04%; χ^2^(1) = 5.58, *p* = 0.018; Cramér’s V = 0.14). Significant differences were also recorded for metal–acrylic FPDs compared to metal–ceramic FPDs (98.41% vs. 92.22%; χ^2^(1) = 5.73, *p* = 0.017; V = 0.14). Regarding the number of elements, biological complications were highly prevalent across all categories, with no statistically significant differences.

Statistically significant differences were observed regarding FPD age: 75.00% of FPDs with 0–5 years, 93.94% with 6–10 years, 97.37% with 11–15 years, 100% with 15–20 years, and 100% of FPDs with >20 years old experienced biological complications. Consistently, when FPD age was analyzed as a continuous variable, patients with biological complications had FPDs of greater age (median 11.00 [8.00–15.00] vs. 9.00 [4.75–9.25]; Mann–Whitney *p* = 0.006; rank-biserial r = 0.40) (Table 4).

#### 3.4.2. Bivariate Associations Between Sociodemographic Factors, FPD Characteristics, and Technical Complications

Technical complications were more prevalent among females compared to males (89.42% vs. 79.59%; χ^2^(1) = 5.44, *p* = 0.020; Cramér’s V = 0.13). No statistically significant differences were observed based on age group (88.24% vs. 86.03%; *p* = 0.725). Statistically significant differences were found according to area of residence: technical complications were more frequent in patients from rural areas compared to those from urban areas (97.37% vs. 82.61%; χ^2^(1) = 10.51, *p* = 0.001; V = 0.19). Significant differences were also recorded for metal–acrylic FPDs compared to metal–ceramic FPDs (92.06% vs. 82.22%; χ^2^(1) = 6.06, *p* = 0.014; V = 0.14). Regarding the number of elements, technical complications were present in 100% of short-span FPDs, 93.75% of extensive FPDs, and 83–87% of medium- and long-span FPDs.

Technical complications associated with FPD age were observed in 75.00% of FPDs with 0–5 years old, 83.33% with 6–10 years, 89.66% with 11–15 years, 89.47% with 15–20 years, and 100% in FPDs older than 20 years (Table 4). In analyses using longevity as a continuous variable, FPDs in the group with technical complications were older than those without complications (median 11.00 [8.00–15.25] vs. 9.00 [7.00–12.00]; *p* = 0.002; rank-biserial r = 0.30), suggesting a wear process that increases over time.

#### 3.4.3. Bivariate Associations Between Sociodemographic Factors, FPD Characteristics, and Aesthetic Complications

Aesthetic complications were highly prevalent across gender and age groups, with no statistically significant differences. In contrast, significant differences were observed by area of residence and FPD type, with higher prevalence in rural patients than urban ones (100% vs. 80.87%; χ^2^(1) = 16.98, *p* < 0.001; Cramér’s V = 0.24). Significant differences were also found for metal–acrylic FPDs compared to metal–ceramic FPDs (98.41% vs. 76.67%; χ^2^(1) = 28.47, *p* < 0.001; V = 0.31), representing a moderate effect.

Regarding the number of elements, aesthetic complications were observed in 100% of short-span and extensive FPDs and 82–85% of medium- and long-span FPDs.

Aesthetic complications associated with FPD age showed statistically significant differences, with prevalence of 58.33% for FPDs aged 0–5 years, 80.30% for 6–10 years, 89.66% for 11–15 years, 97.37% for 15–20 years, and 100% for FPDs older than 20 years. Consistently, analyses of continuous variables confirmed that patients with aesthetic complications were generally older (>45 years; median 58.00 [54.00–63.00] vs. 55.00 [49.00–61.00]; *p* = 0.026; r = 0.21) and had FPDs of greater longevity (12.00 [8.25–16.00] vs. 7.50 [6.00–9.00]; *p* < 0.001; r = 0.52) (Table 4).

### 3.5. Bivariate Associations Between Local Risk Factors and Biological, Technical, and Aesthetic Complications

Biological complications were present in 94.77% of patients and were associated with significantly higher DMFT values compared to patients without biological complications (median 18.0 vs. 15.0; *p* = 0.031), indicating a moderate association between cumulative caries experience and biological outcomes (Table 5).

Technical complications were present in 86.27% FPDs (*n* = 264) and absent in 13.73% FPDs (*n* = 42). The median DMFT values were identical between the two groups (18.0 [15.0–21.0] vs. 18.0 [16.0–21.0]), and the difference was not statistically significant (*p* = 0.404; r = 0.08). These findings suggest that technical complications are largely independent of cumulative DMFT experience and are more strongly influenced by sociodemographic factors and characteristics of the FPDs rather than the DMFT index (Table 5).

Aesthetic complications were recorded in 85.62% FPDs (*n* = 262), while 14.38% reported no aesthetic complications (*n* = 44). Median DMFT values were very similar between the two groups (18.0 [15.0–21.0] vs. 18.0 [14.0–20.0]), and the comparison did not reach statistical significance (*p* = 0.120; r = 0.15). Thus, although the predisposition to caries in this group is consistently high, its influence on aesthetic complications appears limited. Aesthetic complications seem to be more affected by factors such as the patient’s environment, type of material, age of the FPD, oral hygiene, and smoking rather than by the DMFT index (Table 5).

When DMFT was analyzed as a continuous predictor across all types of complications, a clear association was observed only for biological complications. Patients with biological complications defined by gingival recession presented higher DMFT values than those without recession (median 18.0 [15.0–21.0] vs. 15.0 [13.0–18.75]; Mann–Whitney *p* = 0.031; rank-biserial r = 0.32), suggesting a moderate relationship between cumulative caries experience and biological soft-tissue impairment. By contrast, DMFT distributions were comparable between patients with and without technical complications (median 18.0 [15.0–21.0] vs. 18.0 [16.0–21.0]; *p* = 0.404; r = 0.08) and between those with and without aesthetic complications (median 18.0 [15.0–21.0] vs. 18.0 [14.0–20.0]; *p* = 0.120; r = 0.15).

Overall, these findings indicate that the DMFT index is mainly associated with biological complications, while technical and aesthetic complications appear to be driven more by sociodemographic variables, FPD-related characteristics, and local risk factors rather than by DMFT.

All patients with unsatisfactory oral hygiene presented biological complications (100%), compared to patients with satisfactory hygiene (93.10%; χ^2^(1) = 5.39, *p* = 0.020; Cramér’s V = 0.13), showing a statistically significant difference. Regarding “probable” bruxism, no statistically significant differences were observed (94.92% vs. 94.68%; *p* = 0.929). Smokers exhibited a higher frequency of biological complications (94.68%) compared to non-smokers (92.31%; χ^2^(1) = 5.50, *p* = 0.019; V = 0.13), with statistically significant differences. These associations for oral hygiene, bruxism, and smoking are summarized in Table 6.

Unsatisfactory oral hygiene was associated with a higher frequency of technical complications compared to satisfactory hygiene (97.30% vs. 82.76%; χ^2^(1) = 10.01, *p* = 0.002; Cramér’s V = 0.18). Technical complications were more frequent in patients with “probable” bruxism (89.36%) compared to those without bruxism (81.36%; χ^2^(1) = 3.92, *p* = 0.048; V = 0.11). Smoking status was associated with an increased frequency of technical complications (93.55%) compared to non-smokers (81.32%; χ^2^(1) = 9.32, *p* = 0.002; V = 0.17). These associations were statistically significant and are summarized in Table 6.

Unsatisfactory oral hygiene was also associated with a higher frequency of aesthetic complications (97.30%) compared to satisfactory hygiene (81.90%; χ^2^(1) = 10.81, *p* = 0.001; Cramér’s V = 0.19), with statistically significant differences. Regarding “probable” bruxism, no statistically significant differences were observed (83.05% vs. 87.23%; *p* = 0.310). Smoking status was associated with a higher prevalence of aesthetic complications (96.77%) compared to non-smokers (78.02%; χ^2^(1) = 21.07, *p* < 0.001; V = 0.26), with statistically significant differences (Table 6).

All these bivariate analyses outline a coherent pattern across all types of complications. Biological, technical, and aesthetic complications were more prevalent in patients from rural areas and in those with metal–acrylic restorations. Longer FPDs age (clinical function of the FPDs) and a higher number of restoration elements (≥5 elements) were also associated with a higher frequency of complications, particularly technical and aesthetic complications.

Unsatisfactory oral hygiene and smoking status were associated with a higher frequency of biological, technical, and aesthetic complications, whereas “probable” bruxism showed statistically significant differences only in association with technical complications. The DMFT index showed a moderate association with biological complications, with statistically significant differences, but not with technical or aesthetic complications, suggesting that caries predisposition is correlated with the occurrence of biological complications, while environmental factors, type of FPD material, restoration age, oral hygiene, and smoking are more strongly associated with technical and aesthetic complications.

As complications frequently co-occurred—nearly 90% of patients presented with at least two types of complications. It was explored whether the same risk factors were associated with complications across multiple domains. Descriptively, unsatisfactory oral hygiene, smoking, metal–acrylic restorations, rural residence, greater restoration age, and long-span FPDs (≥5 elements) were associated with a higher frequency of complications across multiple domains, consistent with the contrasts observed separately for the biological, technical, and aesthetic domains.

## 4. Discussion

Although the treatment of partial edentulism with implant-supported prosthetic restorations is a frequently used option with a high success [23], treatment with FPDs remains a very good option in a range of clinical situations, such as severe bone resorption or when surgical intervention is contraindicated [24,25]. Currently, FPDs are frequently used in routine practice, especially in younger adults [26]. This is due to their ability to provide good retention, stability, aesthetics, comfort, improved oral functions, long longevity, and because they are considered the best therapeutic option after implant-supported restorations [27]. In a report published by Carvalho in 2019, it was noted that the number of totally edentulous patients in the 30–45 age group decreased significantly [28]; consequently, patients with partial edentulism are encountered in considerable numbers in current practice.

FPDs restore impaired masticatory functions under very favorable conditions [29]. Complications of FPDs can have varied and complex causes, and their effects may be immediate or delayed [27].

The present study represents a further investigation of a previous study [16] and was conducted on a group of 306 patients who presented for treatment due to complications of FPDs. Local risk factors provide the context in which FPD complications develop. The data from the present study confirm this trend.

The DMFT index is the best indicator used to assess oral health and is recognized by the World Health Organization [19].

The DMFT index is used globally as the most important measure for evaluating oral and dental health. Furthermore, it is the primary index employed in epidemiological studies on community oral health. It is calculated as the sum of the number of decayed teeth, missing teeth and filled teeth due to caries [30].

However, the DMFT index varies across different groups according to age, gender, and socioeconomic and educational level [31].

In the present study, statistical analysis of the FPD data highlighted caries predisposition, as assessed by the DMFT index, with higher values observed in the age group > 45 years (mean 18.39 ± 4.89). Several other studies have reported poor oral health in older adults, quantified using DMFT index [28]. Carvalho et al. (2019) showed that among adults from 23 European countries, the mean DMFT score ranged from 6.6 to 17.6 (median 12.1) [28].

A study conducted in Saudi Arabia on a population aged 18–28 years reported a mean DMFT score of 11.56 ± 6.32 [19]. Another study conducted in Iran in 2020 reported a DMFT of 11.0 in individuals aged 35–44 years, with a tendency to increase with age [32]. Hosseinpour Sarmadi M. (2025), in a study conducted in Azar on a population with a mean age of 64.15 ± 2.91 years, recorded a DMFT index of 28.42 ± 6.0, with a mean number of missing teeth of 26.58 ± 8.36 [31].

Alongside the DMFT index, oral hygiene represents an important aspect of oral health. The present study showed that 75.82% of patients had good oral hygiene, reflected by brushing twice daily. In a study conducted by Dabbagh et al. (2020) on a population in Saudi Arabia, concerning values were reported, with 52.3% of patients not brushing even once per day [33]. There appears to be a clear opportunity to improve oral health habits through twice-daily brushing with fluoride toothpaste among adults, as only 33–85% reported performing this practice [28]. Patients often become less diligent in their plaque control efforts after completing prosthetic treatment. Dentists should carefully monitor any signs of declining oral hygiene and intervene so that deficiencies are identified early and patients are appropriately informed [6].

Bruxism is increasingly discussed as a risk factor in dental treatments [6,34], with a prevalence of up to 30% in the population [35].

In 2018, sleep bruxism was defined as masticatory muscle activity during sleep, characterized as rhythmic (phasic) or non-rhythmic (tonic), and not considered a movement or sleep disorder in otherwise healthy individuals. Awake bruxism was defined as masticatory muscle activity during wakefulness, characterized by repetitive or sustained tooth contact and/or bracing or thrusting of the mandible, and is not considered a movement disorder in otherwise healthy individuals [36]. To prevent any misunderstanding of the definition, the 2025 International Consensus updated the definition, describing bruxism as a repetitive masticatory muscle activity, characterized by clenching or grinding of the teeth and/or by bracing or thrusting of the mandible [37].

Currently, several diagnostic options for bruxism are available [38,39,40,41]. In the present study, however, bruxism data were collected using a self-report questionnaire previously used in our studies [21].

In previous studies [21,41], this questionnaire allowed the formation of two groups of 20 subjects classified as “possible” bruxism and without bruxism, who were instrumentally evaluated using the dia-Bruxo device based on bruxism indices. Their clinical and instrumental evaluation confirmed the diagnosis of “definite” bruxism for the 20 subjects initially classified as “possible” bruxism based on self-reporting and also showed that 3 more subjects who were initially classified as without bruxism, presented “definite” bruxism. Therefore, it can be appreciated that there may be a slight underreporting of bruxism using our questionnaire.

In this study, bruxism as a risk factor was reported by 38.56% of patients. Several studies have reported similar prevalence values. In Romania, Fluerasu et al. (2022) recorded comparable results [42]. Cavallo and colleagues (2017) conducted a study on 278 Italian students, reporting a prevalence of 37.9% for awake bruxism and 31.8% for sleep bruxism [28].

Regarding smoking status, 40.52% of patients in the present study were smokers. In a study by Dabbagh et al., 25.8% of patients were smokers [33]. Smoking increases the risk of developing cardiovascular diseases (e.g., ischemic heart disease, peripheral vascular disease, and stroke) [43], lung cancer, and chronic obstructive pulmonary disease, as well as oral cavity conditions such as oral cancer, mucosal lesions, periodontal diseases, and dental caries [44]. The success of FPDs depends on rigorous diagnosis, careful treatment planning, and precise clinical execution, along with patient cooperation. Nevertheless, biological, mechanical, and aesthetic complications remain common and can affect not only the functionality and longevity of the restoration but also the integrity of supporting structures and overall oral health [45].

In the present study, the most frequent complications were biological (94.77%), followed by technical (86.27%) and aesthetic complications (85.62%).

Technical complications have been investigated in numerous studies and have been reported as the most common type of FPD complications [46,47].

In the present study, for biological complications, pain was reported in 45.09% of patients. Pain constitutes one of the primary urgencies in patients with FPDs and should be assessed in terms of location, character, severity, timing, and onset. Various factors can exacerbate, alleviate, or modify pain. In most cases, pain under FPDs originates from the pulp, but it may also occur in endodontically treated teeth restored with full-coverage crowns, in which case the possibility of root fracture should be considered, especially for teeth with reduced strength due to endodontic treatment. In cases of root fracture, both the abutment tooth and the FPD are compromised. The most frequent biological complication in the present study was pain, occurring in 45.09% of patients. Pulpal pathology was observed in 28.10% of patients, and apical periodontitis occurred in 26.16%. Other studies have also evaluated the presence of apical periodontitis (AP) in abutment teeth [48,49]. For vital teeth, AP was reported at a lower frequency (7.19%) compared to endodontically treated abutment teeth (35.2%) [48].

Other studies indicate that the most common cause of biological complications in FPDs is dental caries [6,50,51]. Caries has a multifactorial etiology, with bacteria considered the primary factor, while secondary etiology includes genetic and environmental risk factors [52]. Detection can be particularly challenging, especially in completely radiopaque FPDs. In the present study, cervical caries occurred in 40.2% of patients, consistent with the clinical presentation of FPDs. Occlusal problems, including involvement of opposing teeth with large wear surfaces, dental mobility, percussion sensitivity, and cusp fractures, were observed in 50.32% of cases. In another study, involvement of opposing teeth, exemplified by occlusal surface wear, was reported in 20.4% of cases [53].

Regarding technical complications in the present study, the highest frequency was reported for loss of marginal fit (84.96%), while decementation occurred in 32.67% of cases. Within the category of technical complications, loss of retention (decementation), loss of marginal adaptation, and abutment tooth fracture are attributed to the occurrence of extensive cervical caries, which are in turn related to poor oral hygiene. Cervical caries often develop where marginal fit is deficient, subsequently leading to decementation of the respective crowns [50]. In metal–ceramic FPDs, chipping or fracture of the ceramic is frequently reported, particularly during the first year of function, although some reports indicate that the incidence of this complication may slightly decrease thereafter [54]. Fracture of the veneer was reported in 11.76% of cases, with similar results observed in a study by Pjetursson, 2015, where it occurred in 12.7% of cases over a 3-year follow-up [55]. Within FPDs, a range of loads develops in the form of tilting, torsion, and flexion, determined by the mechanical properties of dental materials, the cross-sectional shape, and the length of the FPD, which are transmitted both to the restoration and the abutment teeth [56]. In the present study, abutment tooth fracture occurred in 15.03% of cases, while fracture of the pontic was less frequent at 5.22%. Other studies have also reported technical complications represented by abutment tooth fracture, with a frequency of 2.1% [57].

Regarding aesthetic complications, aesthetic deficiencies of FPDs were reported in 85.62% of cases, and gingival recession was observed in 94.77%. The high frequency of gingival recession can be explained by knife-edge abutment preparations—a commonly used technique—and the subgingival placement of the cervical margin of the aggregation elements. In a study by Patel, 2014, the frequency of aesthetic complications was 62.8%, represented by aesthetic deficiencies in 61.6% of cases and gingival recession in 7% [12].

In the present study, from the statistical analysis of the data regarding the risk factors of FPD complications, the associations of factors with the greatest impact on the categories of complications resulted. Thus, smoking is the risk factor involved in all categories of complications. The predisposition to caries is associated with smoking in the biological complications (pain, cervical caries, pulpal pathology, apical periodontitis, damage to antagonist teeth). Bruxism characterized by occlusal overload is associated with smoking in the technical complications (veneer fracture, abutment tooth fracture, pontic fracture, loss of retention, loss of marginal adaptation) and poor oral hygiene is associated with smoking in the aesthetic complications (aesthetic deficiency, gingival recession).

The analysis of the prespecified null hypotheses revealed a domain-specific pattern. For biological complications, H0_BIO was not supported for DMFT, oral hygiene status, and smoking, indicating significant associations with biological outcomes, whereas it was supported for “probable” bruxism. Regarding technical complications, H0_TECH was not supported for oral hygiene, smoking, and “probable” bruxism, but was supported for DMFT, suggesting a stronger influence of functional and behavioral factors than caries history. For aesthetic complications, H0_AEST was not supported for oral hygiene and smoking, while it was supported for DMFT and “probable” bruxism. Overall, oral hygiene and smoking consistently influenced all complication domains, whereas DMFT and bruxism showed a more selective, outcome-dependent effect.

The limitations of the present study include the sample size, the lack of knowledge of the initial functional status of the abutment teeth, and the fact that FPDs were fabricated by multiple dental laboratories with structural differences in the materials used, as well as being performed in dental offices by different clinicians. Also, oral hygiene and smoking were evaluated using limited criteria, potentially masking dose–response effects. Furthermore, the study did not include all-ceramic FPD, as those have been increasingly adopted in recent clinical practice.

## 5. Conclusions

The present study evaluated the occurrence of complications in metal–ceramic and metal–acrylic FPDs in relation to a range of risk factors. All types of complications were more frequent in patients from rural areas with older metal–acrylic FPDs.

Caries predisposition, assessed by the DMFT index, and smoking were associated with a higher frequency of biological complications.

Bruxism and smoking were associated with technical complications, while unsatisfactory oral hygiene and smoking were associated with aesthetic complications.

Multiple types of complications were often present within the same FPD.

This study highlights the importance of risk factors such as DMFT, bruxism, smoking, and oral hygiene in the prognosis of FPDs.

## Figures and Tables

**Table 1:** Local risk factors—DMFT index by age group, oral hygiene, bruxism, and smoking.

Variable	Category	No.	Frequency (%)	Characteristics	
				Mean ± SD	*p*-Value
DMFT index	≤45 years	34	11.11%	16.41 ± 4.43	0.024
	>45 years	272	88.89%	18.39 ± 4.89	
Oral hygiene	Satisfactory	232	75.82%	-	-
	Unsatisfactory	74	24.18%	-	
“Probable” bruxism	Yes	118	38.56%	-	-
	No	188	61.44%	-	
Smoking status	Yes	124	40.52%	-	-
	No	182	59.48%	-	

Notes: Percentages are calculated from the total sample (*n* = 306). DMFT values are reported as mean ± standard deviation (SD). The *p*-value is obtained from the Mann–Whitney U test (two-sided) comparing DMFT between age groups, and is reported to three decimals (or as “<0.001” when appropriate).

**Table 2:** Frequency of biological, technical, and aesthetic complications in the study group (*n* = 306).

Type of Complication	No.	Frequency (%)
Biological complications	290	94.77%
Technical complications	264	86.27%
Aesthetic complications	262	85.62%

Percentages are calculated from the total sample (*n* = 306); values are reported to two decimal places.

**Table 3:** Biological, technical, and aesthetic complications of FPDs.

Category	Subcategory	No.	Frequency (%)
Biological complications	Pain	138	45.09%
	Cervical caries	124	40.52%
	Pulpal pathology	86	28.10%
	Apical periodontitis	80	26.14%
	Damage to antagonist teeth	154	50.32%
Technical complications	Veneer fracture	36	11.76%
	Abutment tooth fracture	46	15.03%
	Pontic fracture	16	5.22%
	Loss of retention (decementation)	100	32.67%
	Loss of marginal adaptation	260	84.96%
Aesthetic complications	Aesthetic deficiency	262	85.62%
	Gingival recession	290	94.77%

Percentages are calculated from the total sample (*n* = 306); values are reported to two decimal places.

**Table 4:** Bivariate associations between sociodemographic factors, FPD characteristics, and biological, technical, and aesthetic complications.

		Biological Complications			Technical Complications			Aesthetic Complications		
Predictor	Category	Yes (*n* (%))	No (*n* (%))	*p*-Value	Yes (*n* (%))	No (*n* (%))	*p*-Value	Yes (*n* (%))	No (*n* (%))	*p*-Value
Gender	Female	200 (96.15%)	8 (3.85%)	0.113	186 (89.42%)	22 (10.58%)	**0.020**	182 (87.50%)	26 (12.50%)	0.172
	Male	90 (91.84%)	8 (8.16%)		78 (79.59%)	20 (20.41%)		80 (81.63%)	18 (18.37%)	
Ages	≤45 years	34 (100%)	0 (0.00%)	0.146	30 (88.24%)	4 (11.76%)	0.725	30 (88.24%)	4 (11.76%)	0.646
	>45 years	256 (94.12%)	16 (5.88%)		234 (86.03%)	38 (13.97%)		232 (85.29%)	40 (14.71%)	
Residence	Urban	214 (93.04%)	16 (6.96%)	**0.018**	190 (82.61%)	40 (17.39%)	**0.001**	186 (80.87%)	44 (19.13%)	**<0.001**
	Rural	76 (100%)	0 (0.00%)		74 (97.37%)	2 (2.63%)		76 (100%)	0 (0.00%)	
FPD material	Metal–ceramic	166 (92.22%)	14 (7.78%)	**0.017**	148 (82.22%)	32 (17.78%)	**0.014**	138 (76.67%)	42 (23.33%)	**<0.001**
	Metal–acrylic	124 (98.41%)	2 (1.59%)		116 (92.06%)	10 (7.94%)		124 (98.41%)	2 (1.59%)	
Number of elements	2 elements	8 (100%)	0 (0.00%)	0.344	8 (100%)	0 (0.00%)	0.303	8 (100%)	0 (0.00%)	0.048
	3–4 elements	162 (93.10%)	12 (6.90%)		146 (83.91%)	28 (16.09%)		144 (82.76%)	30 (17.24%)	
	5–7 elements	88 (95.65%)	4 (4.35%)		80 (86.96%)	12 (13.04%)		78 (84.78%)	14 (15.22%)	
	8–16 elements	32 (100%)	0 (0.00%)		30 (93.75%)	2 (6.25%)		32 (100%)	0 (0.00%)	
FPD age	0–5 years	18 (75.00%)	6 (25.00%)	**<0.001**	18 (75.00%)	6 (25.00%)	0.121	14 (58.33%)	10 (41.67%)	**<0.001**
	6–10 years	124 (93.94%)	8 (6.06%)		110 (83.33%)	22 (16.67%)		106 (80.30%)	26 (19.70%)	
	11–15 years	58 (100%)	0 (0.00%)		52 (89.66%)	6 (10.34%)		52 (89.66%)	6 (10.34%)	
	15–20 de years	74 (97.37%)	2 (2.63%)		68 (89.47%)	8 (10.53%)		74 (97.37%)	2 (2.63%)	
	Peste 20 de years	16 (100%)	0 (0.00%)		16 (100%)	0 (0.00%)		16 (100%)	0 (0.00%)	

Note: Percentages were calculated within each category of the predictor. Associations were tested using the two-sided Chi-square test. *p*-values are reported to three decimal places (or “<0.001” where applicable). Bolded values indicate statistical significance at α = 0.05. Effect sizes (Cramér’s V) are summarized in the text.

**Table 5:** Bivariate associations between the DMFT index and biological, technical, and aesthetic complications.

Type of Complication	Complication Status	Frequency (*n* (%))	DMFT Median [IQR]	*p*-Value
Biological	Yes	290 (94.77%)	18.0 [15.0–21.0]	0.031
	No	16 (5.23%)	15.0 [13.0–18.75]	
Technical	Yes	264 (86.27%)	18.0 [15.0–21.0]	0.404
	No	42 (13.73%)	18.0 [16.0–21.0]	
Aesthetic	Yes	262 (85.62%)	18.0 [15.0–21.0]	0.120
	No	44 (14.38%)	18.0 [14.0–20.0]	

**Table 6:** Bivariate associations between oral hygiene, bruxism, smoking status, and biological, technical, and aesthetic complications.

		Biological Complications			Technical Complications			Aesthetic Complications		
Predictor	Category	Yes (*n* (%))	No (*n* (%))	*p*-Value	Yes (*n* (%))	No (*n* (%))	*p*-Value	Yes (*n* (%))	No (*n* (%))	*p*-Value
Oral hygiene	Satisfactory	216 (93.10%)	16 (6.90%)	0.020	192 (82.76%)	40 (17.24%)	0.002	190 (81.90%)	42 (18.10%)	0.001
	Unsatisfactory	74 (100%)	0 (0.00%)		72 (97.30%)	2 (2.70%)		72 (97.30%)	2 (2.70%)	
“Probable” Bruxism	Yes	112 (94.92%)	6 (5.08%)	0.929	168 (89.36%)	20 (10.64%)	0.048	98 (83.05%)	20 (16.95%)	0.310
	No	178 (94.68%)	10 (5.32%)		96 (81.36%)	22 (18.64%)		164 (87.23%)	24 (12.77%)	
Smoking status	Yes	178 (94.68%)	10 (5.32%)	0.019	116 (93.55%)	8 (6.45%)	0.002	120 (96.77%)	4 (3.23%)	<0.001
	No	168 (92.31%)	14 (7.69%)		148 (81.32%)	34 (18.68%)		142 (78.02%)	40 (21.98%)	

Note: Percentages were calculated within each category of the predictor. Associations were tested using the two-sided Chi-square test. *p*-values are reported to three decimal places (or “<0.001” where applicable). Effect sizes (Cramér’s V) are summarized in the text.

## Data Availability

The authors declare that the data of this research are available from the correspondence authors upon reasonable request.

## Abbreviations

The following abbreviations are used in this manuscript:

FPD	Fixed partial dentures
DMS	Dento-maxillary system
DMFT	Decayed, missing and filled teeth
TMD	Temporomandibular disorder
TMJ	Temporomandibular joint
AP	Apical periodontitis
